# Design and Experiment of a PLC-Based Intelligent Thermal Insulation Box for Nursing Piglets

**DOI:** 10.3390/ani14243580

**Published:** 2024-12-11

**Authors:** Bin Sun, Hao Wang, Xuemin Pan, Yaqiong Zeng, Bin Hu, Renli Qi, Dingbiao Long, Shunlai Xu

**Affiliations:** 1 Chongqing Academy of Animal Sciences, Chongqing 402460, China; ww895543212@aliyun.com (B.S.); zengyaqionghai@163.com (Y.Z.); qirenli1982@163.com (R.Q.); longjuan880@163.com (D.L.); xusl@cqaa.cn (S.X.); 2 National Center of Technology Innovation for Pigs, Chongqing 402460, China; gzzx@cqaa.cn (X.P.); hoobean@yeah.net (B.H.)

**Keywords:** temperature control, PLC, behavior, energy saving, suckling piglets

## Abstract

Local heating of the activity area for nursing piglets is crucial for piglet health and the energy efficiency of barn climate control. Traditional heating methods using lamps or covers lack precise control, result in significant energy waste, and cannot be dynamically adjusted according to piglet age or changing environmental temperatures. To address these issues, this study designed a Programmable Logic Controller (PLC)-based thermal insulation box for nursing piglets, utilizing a strip heater instead of the conventional round heating lamp. The design incorporates a movable thermal insulation box that dynamically adjusts the heater’s power based on the real-time monitoring of environmental temperatures and target temperatures specific to piglet age. First, in a controlled laboratory environment, the study tested and compared the spatial temperature uniformity, temporal stability, and power consumption of the new thermal insulation box versus traditional heating methods. Subsequently, animal trials were conducted in a farrowing barn using eight sows with similar farrowing dates as test subjects. The new thermal insulation box was installed in one group, and the traditional heating lamp in the control group. During the trial, ambient temperature, insulation area temperature, piglet behavior, growth performance, and power consumption were recorded. The results showed that compared to the control group, the new system reduced average temperature fluctuations in the insulation area by 31.6% and spatial temperature variation by 78.3%. During animal trials, the average temperatures directly under the heater for the new system versus the control in the insulation area were 39.7 ± 0.2 °C and 30.2 ± 1.4 °C in the first week, 40.9 ± 0.5 °C and 31.6 ± 0.7 °C in the second week, and 32.3 ± 1.5 °C and 28.6 ± 1.7 °C in the third week—significantly (*p* < 0.05) higher in the test group. The new system also reduced total energy consumption by 58.3%. The usage rate of the thermal insulation area by piglets in the test and control groups was 47.5 ± 5.3% and 42.1 ± 6.6%. The daily weight gain of piglets in the test group was 9.8% higher than that of the control group, also significantly (*p* < 0.05) higher. This intelligent thermal insulation box enables precise and dynamic temperature control, reducing heating energy consumption and supporting improved piglet health and welfare.

## Simple Summary

In pig farming, maintaining an optimal temperature for nursing piglets is critical for their health and growth. Traditional localized heating methods, such as heating lamps, often lack precise temperature control, leading to energy waste and suboptimal conditions for piglets as they grow. This study developed a Programmable Logic Controller-based intelligent thermal insulation box that uses a strip heater to automatically adjust its temperature based on the piglets’ age and environmental conditions. We tested the system’s effectiveness by comparing it to traditional heating in terms of temperature stability, energy consumption, and piglet growth. The new system significantly improved the temperature stability, reduced energy consumption by over 50%, and enhanced piglet growth. This intelligent thermal insulation box could provide an effective, energy-saving solution for improving piglet welfare and farming efficiency.

## 1. Introduction

China is the largest pig-farming country in the world, with a global swine inventory reaching 778,109 million head in 2023. The top three countries by inventory are China, the EU (27 countries), and the United States [1]. However, China’s pig productivity remains relatively low, with the market sow year (MSY)—the average number of pigs sold per sow annually—at 14.4, compared to 25 in developed countries [2]. Mortality among nursing piglets is a significant factor affecting MSY [3,4,5], with crushing by the sow being a primary cause of death among nursing piglets [6,7]. Cold stress, due to low environmental temperatures, causes piglets to huddle close to the sow for warmth, significantly increasing the risk of being crushed. In farrowing beds, the optimal temperature for sows is 18–22 °C, whereas the optimal temperature for piglets in the first week of life is 28–32 °C [8]. Therefore, local heating equipment is essential for piglets to prevent cold stress that might cause them to move too close to the sow. Common heating devices include heat lamps and heating pads. Heating pads offer conductive surface heating with lower power and higher heat efficiency, which also reduces diarrhea incidence in piglets [9,10]. However, heating pads come with high material costs and complex installation requirements. On the other hand, heat lamps are a cost-effective and commonly used heating method in large-scale pig farms worldwide but have drawbacks such as uneven temperature distribution and high power consumption [11,12]. In colder regions, the energy consumption for heating can account for half of the total energy cost from farrowing to finishing, with 70% of this energy dedicated to local heating [13]. Some studies [14,15] have shown that as nursing piglets grow, their metabolic rate and body temperature increase, leading to a gradual reduction in their optimal environmental temperature. When using traditional heat lamps for local heating, the fixed power setting results in energy waste and fails to meet the piglets’ changing growth needs [16].

In existing research, Titterington et al. [17,18,19] examined the impact of heat lamp positioning on piglets and sows. Xin et al. [20] designed an energy-saving 175 W heat lamp, comparing it to a traditional 250W lamp. The former showed better piglet performance and a lower failure rate, yet it still used fixed-power heating equipment, lacking precise temperature control for piglets at different ages. Zhang et al. [21,22] designed different optimal temperatures based on piglet growth stages, using their self-developed system to control heat lamp power, demonstrating significant energy savings. However, they did not report the piglet usage rates of the incubator or piglet production performance. Yu et al. [23] developed an automatic temperature control system for a farrowing house, using a PLC (Programmable Logic Controller) for temperature data collection and control. This system maintained the local environment within the optimal temperature range for piglets, with a margin of error of 3.7%. Yet, they did not conduct animal trials, so its effect on piglets remains uncertain. Souza et al. [24] evaluated two control techniques for resistance heating systems (proportional–integral–derivative controllers and thermostats) in terms of the thermal environment, piglet performance, and electricity use. The results showed that the PID-controlled system excelled in power efficiency, thermal comfort, and piglet growth performance, despite the notable lack of data on the incubator usage rates. Zhou et al. [25] improved a piglet heating device using far-infrared carbon fiber heating plates and insulation materials, which significantly increased piglets’ daily feed intake, weight gain, and immunity while reducing diarrhea and energy consumption. However, the economic benefits were not analyzed, making it unclear whether the heating system is cost-effective.

Adjusting the ambient temperature with power-regulated heating equipment can provide a comfortable environment for nursing piglets at different growth stages while reducing energy costs. This study designed an intelligent heating system for nursing piglets, based on a PLC and a PID control algorithm, to compare with traditional piglet heating equipment that lacks temperature control. The study verified energy consumption, temperature stability, piglet growth performance, and behavior between the two systems, aiming to identify an optimal temperature control approach to provide reference for precision temperature regulation in piglet heating devices.

## 2. Materials and Methods

### 2.1. Structural Design of Thermal Insulation Box

**Figure 1:**
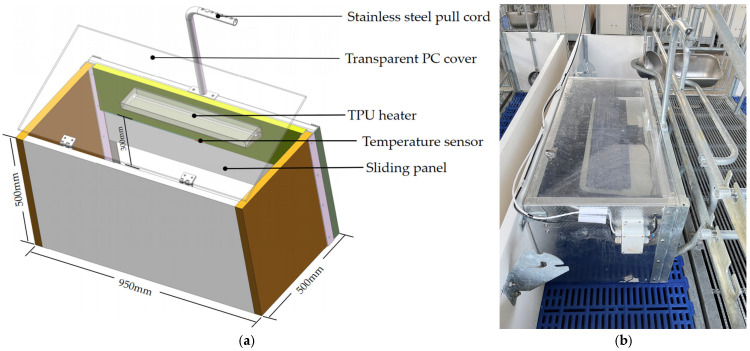
Design drawing of insulation box structure. (**a**) Design diagram. (**b**) Actual view.

As shown in Figure 1, the thermal insulation box designed in this study includes a housing with dimensions of 950 mm in length, 500 mm in width, and 500 mm in height. The main structure is made of PVC (Polyvinyl Chloride) and contains an internal TPU (Thermal Processing Unit) heater (Big herdsman, maximum power 150 W) and a pt100 temperature sensor (accuracy ±0.15 °C) for real-time temperature monitoring and regulation. One side of the housing is designed as an open entry point for the thermal insulation box, with a height of 300 mm. The opening can be adjusted using a sliding panel made of PVC, which is positioned within guide rails on both sides and can move up and down within the slots. A stainless steel pull cord is attached to the top of the sliding panel and can be adjusted to different heights using anchor points on the cord, thereby controlling the degree of the opening. This design facilitates flexible adjustments of the thermal insulation box’s temperature and air circulation according to the piglets’ age and needs. The back panel of the housing includes a transparent polycarbonate (PC) cover for observing piglet conditions, equipped with damped hinges to facilitate quiet, slow closure. This system provides a temperature-controlled enclosure for piglets from birth to weaning, gradually reducing the heat as they grow. The sliding panel helps minimize air turbulence and facilitates routine management tasks such as immunization and tagging. It enables convenient switching between fully and partially enclosed modes, thereby improving the piglet rearing environment.

### 2.2. The Control Logic of the Thermal Insulation Box

**Figure 2:**
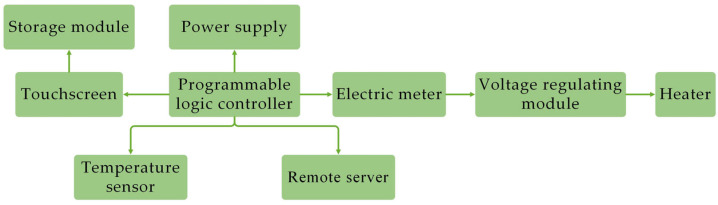
Structure diagram of intelligent insulation system for suckling piglets.

**Figure 3:**
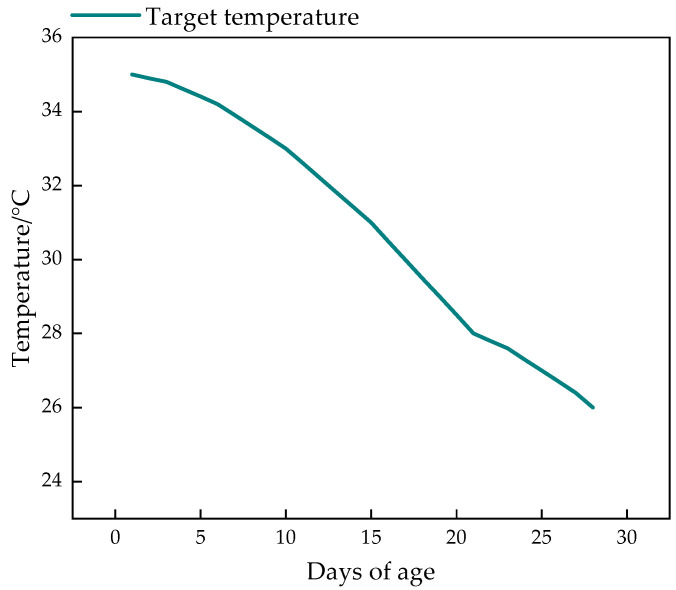
Target temperature curve of insulation box.

In this study, we independently designed an intelligent thermal insulation system for nursing piglets, as shown in Figure 2. The system comprises a touchscreen, temperature monitoring module, PLC control module, storage module, and heater, enabling the precise control of the local temperature around the piglets. To achieve this, this study incorporated age-specific target temperature curves for piglets into the storage module, referencing PIC’s Gilt and Sow Management Guidelines [26] and the Chinese National Standard GB/T 17824.3-2008 Environmental Parameters and Management for Intensive Pig Farms [27], as depicted in Figure 3.

**Figure 4:**
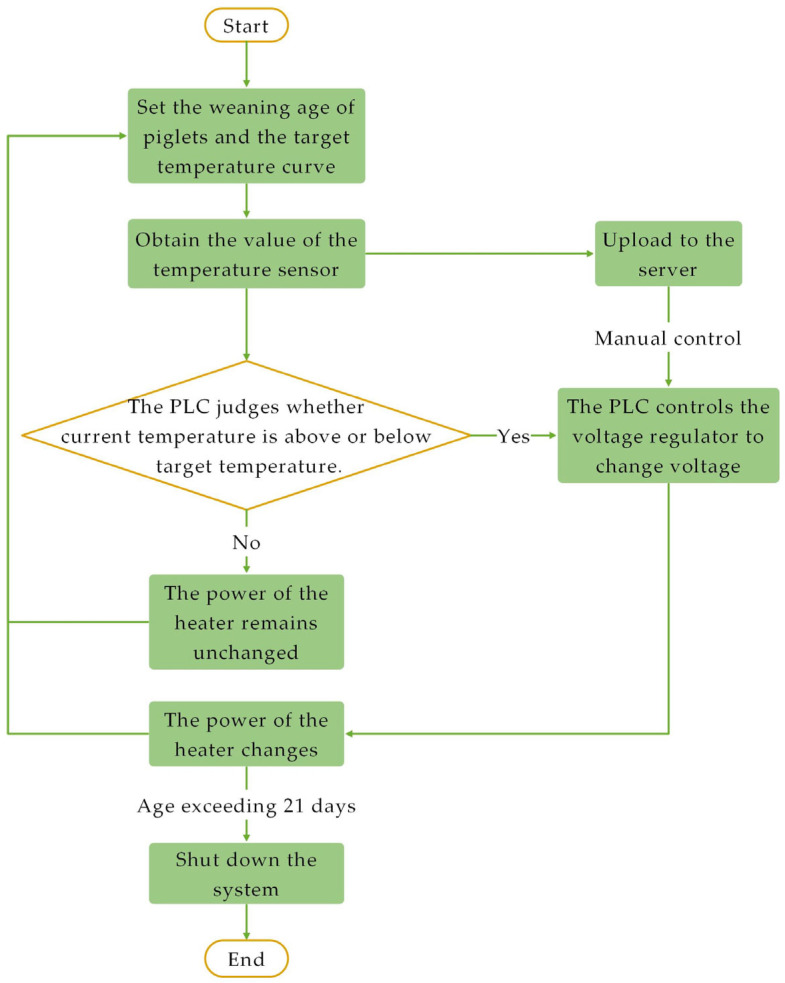
Temperature control logic flowchart.

The system’s control flow is illustrated in Figure 4. Using the touchscreen, users can preset the piglets’ age (e.g., 21 days), the frequency of temperature collection in the thermal insulation box (e.g., once per second), and target temperature values for different ages (ranging from 26 °C to 35 °C) in the storage module. The PLC control module incorporates a PID control algorithm. The temperature monitoring module collects the insulation box temperature t_c_ every second and sends it as a pulse signal to the temperature transmitter. The transmitter converts this pulse signal into a 485 analog signal, which is then transmitted back to the PLC control module.

During temperature adjustment, the PLC control module adjusts the heater output by comparing the current insulation box temperature t_c_ with the target temperature t_d_. If t_c_ is higher than t_d_, the PLC sends a voltage reduction signal to the voltage regulation module, which decreases the heater’s output voltage by 1% at each step, starting from the rated 220 V. If t_c_ is lower than t_d_, the PLC sends a voltage increase signal, raising the output voltage by 1% each time.

The system calculates age daily at midnight, and after reaching 21 days, the PLC control module automatically halts the heater. The storage module records and exports data on piglet age, target temperature, and temperature variations. The system also supports remote control and data upload. Data from the storage module can be transmitted to a remote server via the PLC, allowing users to view data and adjust parameters remotely on a computer or mobile device. Once new parameters are sent to the PLC, it adjusts the heater power accordingly, achieving precise temperature control.

### 2.3. Performance Test Experiment

When the stability and uniformity of environmental temperature are poor, it can easily cause stress in pigs [28,29], impacting their health. To verify whether the internal temperature distribution of the experimental and control equipment was spatially uniform and if temperature fluctuations over time were severe, a temperature distribution variance measurement experiment was designed. The experiment was conducted in a constant-temperature laboratory set at 20 °C, and temperature monitoring was performed using the HOBO MX2301A (Onset Computer Corporation, Bourne, MA, USA), which has a temperature monitoring accuracy of ±0.2 °C and a range of −40 °C to 70 °C.

The experimental group consisted of the thermal insulation box designed in this study, while the control group featured a traditional thermal cover measuring 1250 mm in length and 484 mm in width. The cover was equipped with a heat lamp (250 W, Red, InterHeat Inc., Seongnam, Republic of Korea) installed in a central mounting slot 0.5 m above the ground.

**Figure 5:**
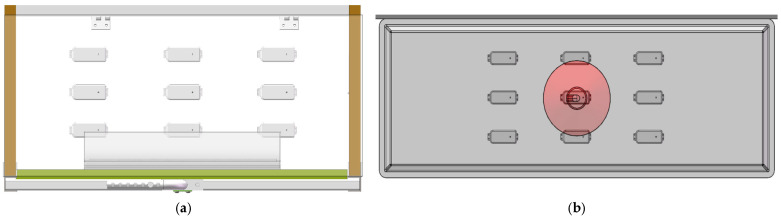
Performance testing sensor layout diagram. (**a**) Experimental group. (**b**) Control group, the red circle is the lampshade of the heat lamp.

The experimental procedure was as follows:

1. Ensure that the heat lamps in both the experimental and control groups are turned off before the experiment begins, and that the temperature within the thermal insulation areas and the test environment is roughly the same.
2. Set the target temperature T_t_ of the experimental group to 35 °C and 28 °C, and correspondingly set the control group to full and half power. Simultaneously activate the smart thermal insulation box and the traditional heat lamp, allowing them to run continuously for 4 h. During this period, monitor the temperature T_n_ in the insulation area and T_w_ in the ambient environment using temperature sensors. Nine measurement points were arranged within both the insulation area of the box and the thermal cover (see Figure 5 for the distribution layout), with one measurement point in the external environment. Each measurement point was located 0.3 m from the ground, and data from each point were recorded every minute. Simultaneously, the temperature T_s_ from the built-in probe of the insulation box controller in the test group was recorded.
3. After 4 h of operation, turn off both the experimental group’s insulation box and the control group’s heat lamp. Export the data for T_s_, T_n_, and T_w_, which correspond to the internal probe temperature, insulation area temperature, and ambient temperature, respectively.

Temperature fluctuation: Starting from the time when T_s_ first reaches or exceeds T_t_, temperature data from 18 measurement points in the thermal insulation areas of both the experimental and control groups are collected over a 2 h period. These data are then used to calculate the temperature fluctuations in both the experimental and control groups, as shown in Equations (1)–(3).

(1) $$T_{avni}=\frac{\sum_{j=1}^{9}T_{nij}}{9},$$

(2) $$T_{avns}=\frac{\sum_{i=1}^{120}T_{avni}}{120},$$

(3) $$T_{dt}=\sqrt{\frac{\sum_{i=1}^{120}{\left(T_{avni}-T_{avns}\right)}^{2}}{120}},$$

Here, T_nij_ represents the temperature reading of the j-th sensor at the i-th minute, T_avni_ is the average temperature of the nine measurement points in both the experimental and control groups at the i-th minute within 2 h, and T_avns_ is the average of all 120 T_avni_ values over the 2 h period. T_dt_ denotes the standard deviation, representing the temperature fluctuation value. Using this temperature fluctuation value, the temperature fluctuation reduction rate can be calculated as shown in Equation (4).

(4) $$T_{dzct}=\frac{T_{dtc}-T_{dt}}{T_{dtc}}\times 100\%,$$

Here, T_dtc_ represents the temperature fluctuation of the control group, T_dt_ represents the temperature fluctuation of the experimental group, and T_dzct_ represents the temperature fluctuation reduction rate.

Temperature deviation: Starting from the time when T_s_ first reaches or exceeds T_t_, temperature data from 18 measurement points in the thermal insulation areas of both the experimental and control groups are collected over a 2 h period. These data are then used to calculate the temperature deviation for the experimental and control groups, as shown in Equations (5)–(7).

(5) $$T_{avnj}=\frac{\sum_{i=1}^{120}T_{nij}}{120},$$

(6) $$T_{avnu}=\frac{\sum_{j=1}^{9}T_{avnj}}{9},$$

(7) $$T_{ds}=\sqrt{\frac{\sum_{j=1}^{9}{\left(T_{avnj}-T_{avnu}\right)}^{2}}{9}},$$

Here, T_avnj_ is the mean temperature of the j-th measurement point over 2 h, based on a total of 120 values of T_nij_. T_avnu_ is the average of T_avnj_ values across the 9 measurement points, and T_ds_, the standard deviation of T_avnu_, represents the temperature deviation. The temperature deviation reduction rate can then be calculated, as shown in Equation (8).

(8) $$T_{dzcs}=\frac{T_{dsc}-T_{ds}}{T_{dsc}}\times 100\%,$$

Here, T_dsc_ represents the temperature deviation in the control group, T_ds_ represents the temperature deviation in the experimental group, and T_dzcs_ is the temperature deviation reduction rate.

Temperature difference T_d_: Beginning from the time when the internal probe temperature T_s_ of the thermal insulation box first reaches or exceeds the target temperature T_t_, the average temperature T_av2_ of the measurement point T_2_ directly below the heater inside the insulation box over the next 2 h is calculated. The temperature difference T_d_ is then calculated between T_av2_ and the target temperature T_t_, as shown in Equations (7)–(10):

(9) $$T_{av2}=\frac{\sum_{i=1}^{120}T_{2i}}{120},$$

(10) $$T_{d}=\left|T_{av2}-T_{t}\right|,$$

### 2.4. Animal Trials

The animal feeding trial was conducted at the Shuanghe Pig Nutrition and Environmental Control Base in Rongchang District, Chongqing, from March to April 2024. The farrowing house unit used in the trial measures 44.5 m × 9.3 m × 2.75 m and employs a positive-pressure ventilation system on the roof, equipped with five positive-pressure fans. Additionally, the side walls were fitted with five positive-pressure cooling fans, which are typically activated during summer. However, these side fans were not in operation during the winter trial period. During the trial period, staff were on-site all day to manually the control fan operation in response to indoor temperature conditions, maintaining a stable indoor temperature of approximately 19 °C in the farrowing unit. The farrowing unit layout includes two rows with three walkways and 40 stalls, with each farrowing bed measuring 2.4 m × 1.8 m. There were four sows in the experimental group with a total of 39 live piglets, and four sows in the control group with a total of 54 live piglets. Each group was equipped with four heating devices: the experimental group’s heaters were fitted with an automatic temperature control system, allowing them to adjust the power based on the target temperature, while the control group’s heating lamps operated at a fixed power setting. Throughout the trial, both the experimental and control group heating devices were operated continuously. Data collected during the trial included internal and external temperatures of heating devices, piglet behavior data, piglet production performance, and device energy consumption.

#### 2.4.1. Collection of Ambient Temperature Data

**Figure 6:**
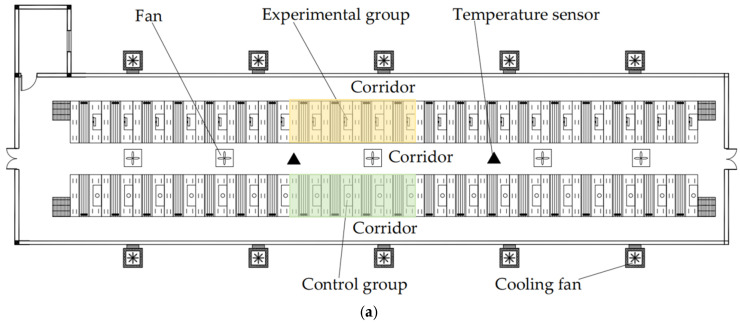

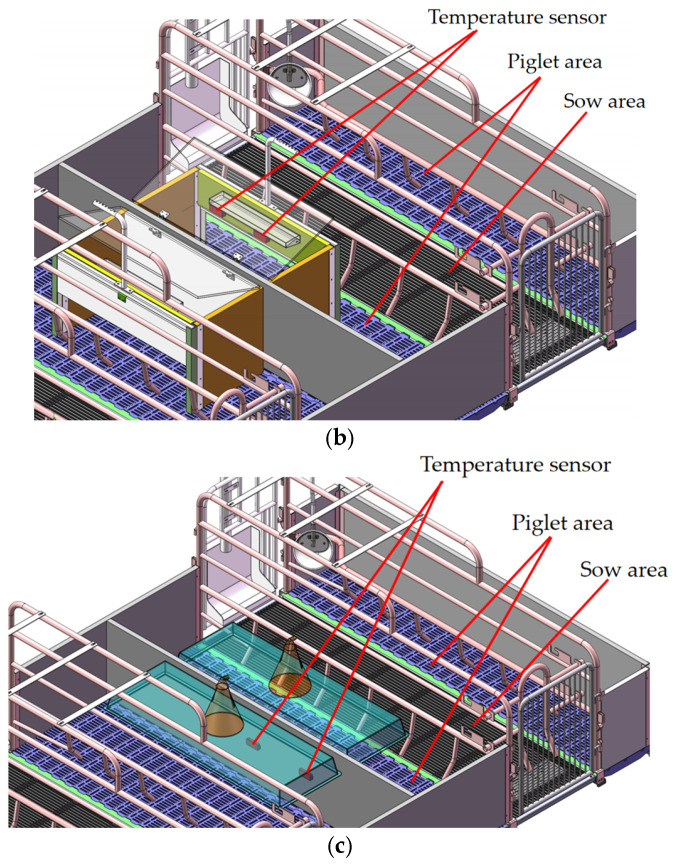
Distribution of farrowing rooms and their sensors. (**a**) Experimental pig house and indoor sensor distribution; (**b**) distribution of farrowing beds and sensors in the experimental group; (**c**) distribution of farrowing beds and sensors in the control group.

The experimental setup, including the control and experimental groups and their sensor placements, is shown in Figure 6. Due to limited experimental materials, a total of 4 + 4 farrowing beds were selected for the experiment. To compare the differences between internal temperatures in the experimental and control groups and the indoor temperature, two indoor temperature measurement points were evenly set in the feeding aisle at a height of 0.9 m above the ground. Temperature data were collected using the HOBO MX2301A sensor from the Bourne, MA, USA, and data were exported every half-month to prevent data from being overwritten.

As shown in Figure 6b,c, sensor placements within the experimental and control group equipment differed. In the experimental group, two HOBO MX2301A temperature sensors were placed on the right side of the thermal insulation box at a height of 0.3 m, with an additional built-in temperature probe located in the center of the right side, aligning with the height of the other temperature sensor. In the control group, two temperature measurement points were set on the left side of the thermal insulation hood, also at a height of 0.3 m above the ground. To ensure the scientific rigor of the experiment, measurement points in the experimental and control groups were not set in exactly the same locations. This distinction is due to the control group’s insulation hood being closer to the internal left side back panel area of the equipment, where temperature changes are minimal and insulation is relatively more effective. In contrast, the temperature measurement points in the experimental group were placed on the right side of the thermal insulation box, a location with greater temperature variation that is more affected by external factors. This setup was intended to compare the performance of the thermal insulation equipment under different conditions, thereby validating the robustness of the experimental group by comparing its most challenging conditions against the control group’s optimal conditions.

#### 2.4.2. Collection of Behavior Data

A Hikvision dome camera (model DS-2DC2C40IY-DE(F1), Hangzhou, China) was installed on the ceiling directly above each farrowing bed to record piglet behavior. Video footage was collected for three periods, i.e., the first three days after birth, days 8–10, and days 15–17, totaling 9 × 24 h of recordings. The footage was processed using the FFmpeg (version N-111976-gb97ac6b3df-20230907) software program, capturing one image every 5 min, resulting in 144 images every 24 h. The images were then analyzed to record the number of piglets outside the thermal insulation box at each captured time point, which can obtain the usage rate of the experimental and control equipment.

#### 2.4.3. Production Performance Analysis Method

The indicators used to analyze production performance included the litter size, born alive, birth mass, weaning mass, and average daily weight gain of the piglets. Due to the practice of litter mixing shortly after the sows give birth, the total litter size was calculated as the sum of the number of piglets born to the sow plus the number added through mixing. The birth mass was calculated as the litter size minus the number of stillbirths.

#### 2.4.4. Analysis Methods of Energy Consumption and Economic Benefits

The experimental group’s control system automatically recorded the heater’s power consumption and output every 1 min, while the control group manually recorded the electric meter reading at 9 a.m. each day. By tracking the daily power consumption of both the experimental and control groups, the average daily power consumption and average power of each heating lamp were calculated. The formula for calculating average power consumption is shown in Equation (11).

(11) $$W=\frac{\sum_{k=1}^{n}\frac{\sum_{i=1}^{d}W_{i}^{k}}{d}}{n},$$

where W represents the average daily power consumption of each heating lamp, $W_{i}^{k}$ is the power consumption of the k-th heating lamp on the i-th day during the experiment, d is the number of experimental days, and n is the number of heating lamps. The formula for calculating average power is shown in Equation (12).

(12) $$P=\frac{\sum_{i=1}^{d}\sum_{j=1}^{1440}\sum_{k=1}^{n}P_{i,j,k}}{1440\times d\times n},$$

where P represents the daily average power, $P_{i,j,k}$ is the output power of the k-th heating lamp at the j-th recording on the i-th day, and 1440 indicates that output power is recorded 1440 times per day. The method for calculating the energy savings of the experimental group compared to the control group is shown in Equation (13).

(13) $$W_{save}=n\times (W_{c}-W_{e})\times d,$$

where W_save_ represents the total energy saved during the experiment, and W_c_ and W_e_ are the daily average power consumption per heating lamp for the control and experimental groups, respectively.

## 3. Results and Discussion

### 3.1. Performance Test Results

The temperature difference, temperature deviation, and temperature fluctuation indicators for the area below the devices in the experimental and control groups were calculated according to Formulas (1)–(10) under pig-free conditions. The calculation results are shown in Table 1.

As shown in Table 1, when the target temperature was 35 °C, the average temperature difference between the experimental group and the target temperature was 2.63 °C, while for the control group, it was 3.4 °C. The temperature fluctuation within the thermal insulation box of the experimental group was 0.16 °C, compared to 0.19 °C in the control group, indicating a temperature fluctuation reduction rate of 14.9% in the experimental group relative to the control group. The temperature deviation within the thermal insulation box of the experimental group was 1.26 °C, significantly lower than that of the control group at 7.57 °C (*p* < 0.01), with a temperature deviation reduction rate of 83.3%. The power consumption of the experimental group was 0.12 kW∙h, significantly lower than the control group at 0.24 kW∙h (*p* < 0.01), with a power-saving rate of 51.1% relative to the control group.

When the target temperature was 28 °C, the average temperature difference between the experimental group and the target temperature was 1.26 °C, while it was 4.62 °C in the control group. The temperature fluctuation within the box of the experimental group was 0.11 °C, compared to 0.23 °C in the control group, showing a temperature fluctuation reduction rate of 48.2% for the experimental group. The temperature deviation within the box was 0.80 °C for the experimental group, significantly lower than the control group’s 3.00 °C (*p* < 0.05), with a temperature deviation reduction rate of 73.2%. The power consumption of the experimental group was 0.07 kW∙h, compared to 0.09 kW∙h for the control group, with a power-saving rate of 16.67%.

Based on the experiments at both target temperatures, the average temperature difference for the experimental group was 1.9 °C, with an average temperature fluctuation of 0.13 °C and a temperature deviation of 1.0 °C. For the control group, the average temperature difference was 4.0 °C, with an average temperature fluctuation of 0.21 °C and a temperature deviation of 5.3 °C. Although the temperature fluctuation in the experimental group was not significantly different from that in the control group, its average value was lower than that of the control group, showing a decreasing trend. The experimental group showed a reduction rate of 31.6% in temperature fluctuation and 78.3% in temperature deviation relative to the control group.

### 3.2. Results of Animal Trials

#### 3.2.1. Indoor Temperature and Temperature Under Thermal Insulation Equipment

**Figure 7:**
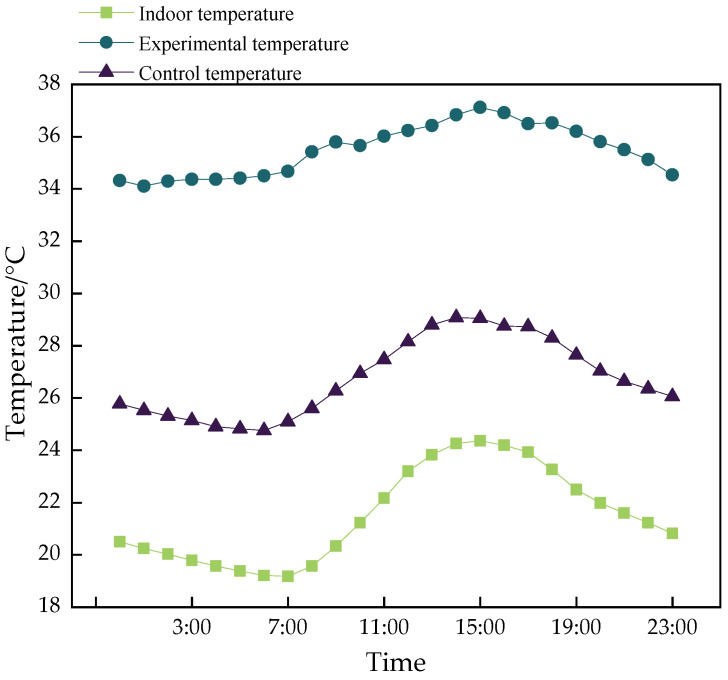
Average ambient temperature.

The average daily indoor temperature and temperature variations in the experimental and control groups during the trial period are shown in Figure 7. The figure shows that the minimum indoor temperature was 19.1 °C at 7:00, and the maximum was 24.3 °C at 15:00, with an average of 21.5 °C. The control group displayed a similar temperature trend to the indoor environment, with a minimum of 24.8 °C at 6:00 and a maximum of 29.0 °C at 14:00, averaging 26.8 °C. For the test group, the minimum temperature was 34.1 °C at 1:00, the maximum was 37.1 °C at 15:00, and the average was 35.4 °C. The temperature range in the experimental group was 3.0 °C, compared to 4.2 °C in the control group. This smaller range in the experimental group indicates reduced temperature fluctuation, consistently with the results discussed in Section 3.1, where the experimental group showed a trend toward decreased temperature fluctuation.

As shown in Table 2, both the experimental and control groups exhibited elevated temperatures within the thermal insulation equipment during the second week post-birth, though temperatures in both groups generally displayed a decreasing trend over the first three weeks. The elevated temperatures in the second week may have been caused by a sudden increase in external environmental temperatures. Throughout the trial period, the temperature inside the experimental group’s equipment remained above 30 °C, while in the control group, it ranged from 23 °C to 32 °C. From days 1 to 21 after birth, both the center and side temperatures within the experimental group’s equipment were significantly (*p* < 0.05) higher than those in the control group’s equipment. Compared with the results of studies [8,21], the experimental group in this study exhibited higher temperatures in the thermal insulation area, which may be attributed to the higher average farrowing room temperature during the experimental period in this study compared to the farrowing room temperatures in [8,21].

#### 3.2.2. Piglet Behavior

To investigate the impact of temperature under the thermal insulation equipment on piglets in the experimental and control groups, this study conducted a statistical analysis of piglet behavior data from both groups, yielding the proportion of piglets active under the equipment at various stages, which represents the usage rate of equipment. The results are shown in Table 3.

As shown in Table 3, the usage rate of the thermal insulation box by piglets during the first week after birth was the highest across all observation periods, with similar rates during both day and night. In the following two weeks, as the piglets aged and their thermoregulation abilities improved, their use of the thermal insulation box gradually decreased, and they were more inclined to be active under the box at night. This pattern is generally consistent with the findings in [30]. Additionally, as piglets age, they tend to transition from warmer areas to cooler ones for resting, as suggested in [31]. This behavior may also be due to reduced available space inside the thermal insulation box as the piglets grew larger, coupled with their increased exploratory behavior, prompting them to spend more time outside the box. Comparing behavior statistics between the experimental and control groups, the experimental group’s piglets consistently used the thermal insulation box more frequently than the control group during the first three weeks after birth. The difference was most pronounced in the second week, with the experimental group showing a daytime usage rate of 38.0%, a nighttime usage rate of 52.9%, and a total of 45.9%, compared to the control group’s 33.7% during the day, 39.3% at night, and a total of 35.3%. Although the difference between the experimental and control groups was not statistically significant, from a practical farming perspective, the experimental group may have a certain advantage in attracting piglets to use the thermal area, which could have potential value in improving the rearing environment and enhancing farming efficiency.

#### 3.2.3. Piglet Production Performance

The production performance of piglets in the experimental and control groups is shown in Table 4. The differences in litter size, born alive, birth mass, and weaning mass of piglets between the experimental and control groups were not significant (*p* > 0.05). This may be due to environmental and genetic factors. Firstly, the piglets were raised under similar environmental conditions, including temperature, humidity, and feed nutrition, which may have masked any potential differences. Secondly, the genetic backgrounds of the piglets in the experimental and control groups may be similar, leading to no significant differences in litter size or other growth traits, as genetics significantly influence growth traits, especially in experiments conducted within the same population where individual genetic differences are minimal [32]. The average daily weight gain of the experimental group, at 254.3 g per piglet, was significantly higher (*p* < 0.05) than that in the control group, which averaged 231.5 g per piglet, indicating that the equipment used in the experimental group had a beneficial effect on the growth performance of the piglets. It is worth noting that the average number of born alive was 9.7 in the experimental group and 13.5 in the control group. Therefore, the higher average daily weight gain observed in the experimental group might be attributed to reduced competition for milk. Further studies are needed to confirm this.

#### 3.2.4. Energy Consumption Analysis of Incubator

Comparing the energy consumption of the thermal insulation boxes in the experimental and control groups, the power and electricity usage of both groups are shown in Table 5. During the first three weeks after the piglets were born, the average power and daily electricity consumption of the experimental group were significantly lower (*p* < 0.01) than those of the control group. In the first week, each thermal insulation box in the experimental group saved an average of 2.7 kW∙h per day, with the same savings in the second week, and 5.0 kW∙h per day in the third week. Over the 21-day nursing period, the experimental group saved an average of 3.5 kW∙h per day, with an energy savings rate of 58.3%. These findings are consistent with the results of [8], which also demonstrated that adjusting the heating power of thermal equipment according to the age of piglets can effectively reduce energy consumption. Furthermore, compared to the daily electricity consumption reported in [8], the experimental group in this study exhibited even lower daily consumption. It is worth noting that the energy consumption data in this study were derived under specific environmental conditions, with barn temperature potentially being one of the influencing factors. As the environmental conditions in this study differed from those in [8], further research is needed to verify this conclusion. Given the agricultural electricity price of 0.568 CNY/(kW∙h) at the experimental pig farm and based on Equations (7) and (8), the electricity savings for raising one litter of piglets over 21 days amounted to 72.7 kW∙h, saving CNY 41.3 in electricity costs.

## 4. Conclusions

(1) In performance testing under pig-free conditions, the average temperature difference in the experimental group was 1.9 ± 0.7 °C, with an average temperature fluctuation of 0.13 ± 0.1 °C and an average temperature deviation of 1.0 ± 0.1 °C. In the control group, the average temperature difference was 4.0 ± 1.1 °C, average temperature fluctuation was 0.21 ± 0.1 °C, and average temperature deviation was 5.3 ± 0.4 °C. Compared to the control group, the experimental group showed a 31.6% reduction in temperature fluctuation and a significantly (*p* < 0.05) reduced temperature deviation of 78.3%.
(2) In the animal husbandry trial under pig-rearing conditions, the average temperature in the experimental pig house during the trial was 21.4 °C. The average temperature directly under the equipment in the experimental and control groups during the first week after the piglets were born was 39.7 ± 0.2 °C and 30.2 ± 1.4 °C, respectively; in the second week, it was 40.9 ± 0.5 °C and 31.6 ± 0.7 °C; and in the third week, it was 32.3 ± 1.5 °C and 28.6 ± 1.7 °C. The temperature in the experimental group was significantly higher (*p* < 0.05) than in the control group. Meanwhile, the usage rate of the thermal insulation box in the experimental group was 44.8 ± 4.1% during the day and 50.0 ± 8.2% at night, totaling 47.5 ± 5.3%. In the control group, the usage rate of the thermal insulation box was 39.4 ± 7.0% during the day and 45.6 ± 6.8% at night, totaling 42.1 ± 6.6%. Although the difference in usage rates between the two groups was not statistically significant, the experimental group still showed a positive trend in the utilization of the piglet warming area.
(3) In the first week after birth, each thermal insulation box in the experimental group saved an average of 2.7 kW∙h per day; in the second week, the daily savings remained 2.7 kW∙h; and in the third week, it was 5.0 kW∙h. During the 21-day weaning period, each thermal insulation box saved an average of 3.5 kW∙h per day, with an energy-saving rate of 58.3%. Under experimental conditions, raising one litter of piglets (over 21 days) saved a total of 72.7 kW∙h in electricity, reducing costs by CNY 41.3.
(4) Under the current experimental conditions, the average daily weight gain in the experimental group was 254.3 g/head, while in the control group it was 231.5 g/head, indicating a 9.8% improvement in the experimental group (*p* < 0.05). This result clearly demonstrates the significant advantage of the experimental group in promoting piglet growth by providing a more favorable environment for their development.

Based on the comprehensive results of this study, the newly designed thermal insulation box demonstrates significant advantages in temperature control performance, energy-saving efficiency, and promoting piglet growth. It effectively improves the environmental quality and economic benefits during piglet rearing. However, due to the relatively small sample size and the lack of adjustment in the number of piglets per litter between the experimental and control groups, the findings may have certain limitations. Future research will focus on increasing the sample size and optimizing the experimental design to more precisely investigate the practical effectiveness of this thermal insulation box in piglet farming, providing more scientific and effective temperature control solutions for piglet rearing.

## Figures and Tables

**Table 1:** Calculation results of performance testing indicators.

Indicators	Target Temperature 35 °C		Target Temperature 28 °C	
	Experimental Group	Control Group	Experimental Group	Control Group
Temperature difference/°C	2.63 ± 0.9	3.4 ± 1.4	1.26 ± 0.5	4.62 ± 2.5
Temperature fluctuation/°C	0.16 ± 0.1	0.19 ± 0.1	0.11 ± 0.1	0.23 ± 0.1
Temperature deviation/°C	1.26 ± 0.1 A ^1^	7.57 ± 0.3 B	0.80 ± 0.1 a	3.00 ± 1.1 b
Power consumption/(kW∙h)	0.12 ± 0.1 A	0.24 ± 0.1 B	0.07 ± 0.1 a	0.09 ± 0.1 b

^1^ Different lowercase letters indicate a significant difference between treatments (within the same row) at the 0.05 level (*p* < 0.05), while different uppercase letters indicate a highly significant difference between treatments (within the same row) at the 0.01 level (*p* < 0.01), the same as below.

**Table 2:** Average temperature under thermal insulation equipment.

Days of Age	External Temperature/°C	Central Temperature in the Insulation Area/°C		Side Temperature in the Insulation Area/°C	
		Experimental Group	Control Group	Experimental Group	Control Group
1~7	21.1 ± 0.7	39.7 ± 0.2 a ^1^	30.2 ± 1.4 b	37.7 ± 0.4 a	23.7 ± 0.7 b
7~14	22.3 ± 0.4	40.9 ± 0.5 a	31.6 ± 0.7 b	38.6 ± 0.3 a	25.6 ± 0.4 b
14~21	22.1 ± 0.7	32.3 ± 1.5 a	28.6 ± 1.7 b	31.6 ± 1.3 a	26.3 ± 0.6 b

^1^ Different lowercase letters indicate a significant difference between treatments (within the same row) at the 0.05 level (*p* < 0.05).

**Table 3:** Usage rate of thermal insulation boxes for piglets at different stages.

Days of Age	Experimental Group/%			Control Group/%		
	Daytime	Nighttime	Sum	Daytime	Nighttime	Sum
1~7	63.7 ± 8.5	60.2 ± 9.3	62.0 ± 8.4	57.0 ± 7.0	57.8 ± 7.5	57.4 ± 6.9
7~14	38.0 ± 5.7	52.9 ± 7.3	45.9 ± 2.8	33.7 ± 6.4	39.3 ± 12.6	35.3 ± 8.1
14~21	32.6 ± 8.6	36.7 ± 13.6	35.0 ± 10.9	27.5 ± 9.0	39.6 ± 8.0	33.5 ± 8.5
1~21	44.8 ± 4.1	50.0 ± 8.2	47.5 ± 5.3	39.4 ± 7.0	45.6 ± 6.8	42.1 ± 6.6

**Table 4:** Piglet production performance.

Indicators	Experimental Group	Control Group
Litter size/head	11.0 ± 2.5	15.0 ± 1.5
Born alive/head	9.7 ± 0.4	13.5 ± 0.8
Birth mass/(kg·head^−1^)	1.2 ± 0.1	1.5 ± 0.1
Weaning mass/(kg·head^−1^)	8.1 ± 0.4	7.7 ± 0.2
Average daily gain/(g·head^−1^·d^−1^)	254.3 ± 12.0 a ^1^	231.5 ± 7.0 b

^1^ Different lowercase letters indicate a significant difference between treatments (within the same row) at the 0.05 level (*p* < 0.05).

**Table 5:** Energy consumption of thermal insulation equipment.

Days of Age	Average Power/W		Average Daily Power Consumption/(kW∙h∙d^−1^)	
	Experimental Group	Control Group	Experimental Group	Control Group
1~7	100.0 ± 0.0 A	250 ± 0.0 B	3.3 ± 0.0 A	6.0 ± 0.0 B
7~14	99.7 ± 0.4 A	250 ± 0.0 B	3.3 ± 0.1 A	6.0 ± 0.0 B
14~21	52.3 ± 0.2 A	250 ± 0.0 B	1.0 ± 0.4 A	6.0 ± 0.0 B
1~21	84.0 ± 0.1 A	250 ± 0.0 B	2.5 ± 0.1 A	6.0 ± 0.0 B

Different uppercase letters indicate a highly significant difference between treatments (within the same row) at the 0.01 level (*p* < 0.01).

## Data Availability

The original contributions presented in the study are included in the article, further inquiries can be directed to the corresponding author.

## References

[1] Zhang H., Wang L., Chen Z., Huang X., Chen N. (2024). Development of Global Pig Industry in 2023 and Trends in 2024. Swine Ind. Sci..

[2] Yang Y. (2022). Research on the Development and Application of the Intelligent Heating System for Piglets in the Farrowing House. Master’s Thesis.

[3] Ma C.W., Miao X.W. (2005). Agricultural Bio-Environmental Engineering.

[4] Kim T. (2016). Determinants of the marketed-pig per sow per year for decision-making of piggery entrepreneur in South Korea. Int. J. Adv. Res.

[5] Yang Y., Deng M., Chen J., Zhao X., Xiao K., He W., Qiu X., Xu Y., Yin Y., Tan C. (2021). Starch supplementation improves the reproductive performance of sows in different glucose tolerance status. Anim. Nutr..

[6] Wang L. (2016). A Brief Discussion on the Rearing Management of Nursing Piglets in Large-Scale Pig Farms. Shandong J. Anim. Sci. Vet. Med..

[7] Kielland C., Wisløff H., Valheim M., Fauske A.K., Reksen O., Framstad T. (2018). Preweaning mortality in piglets in loose-housed herds: Etiology and prevalence. Animal.

[8] Wang M., Ren F., Zang J., Chen Z., Hao W., Zhang X., Liu J. (2019). Environmental control and energy saving effect of heat lamp with variable power heating for piglets. Trans. Chin. Soc. Agric. Eng. (Trans. CSAE).

[9] Morrison W.D., Bate L.A., McMillan I., Amyot E. (1987). Operant heat demand of piglets housed on four different floors. Can. J. Anim. Sci..

[10] Su W., Gong T., Jiang Z., Lu Z., Wang Y. (2022). The role of probiotics in alleviating postweaning diarrhea in piglets from the perspective of intestinal barriers. Front. Cell. Infect. Microbiol..

[11] Guan L. (2020). Design and Determination of Numerically Controlled Pen and Its Growth Performance Influence on Piglets. Master’s Thesis.

[12] Lane K.J. (2019). Heat Lamps and Heat Mats in the Farrowing House: Effect on Piglet Production, Piglet and Sow Behavior and Energy Usage. Master’s Thesis.

[13] Barber E.M., Classen H.L., Thacker P.A. (1989). Energy use in the production and housing of poultry and swine—An overview. Can. J. Anim. Sci..

[14] Farm Energy: Conserving Energy by Using Localized Heating in Swine Housing. http://works.bepress.com/jay_harmon/8/.

[15] Close W.H., Stanier M.W. (1984). Effects of plane of nutrition and environmental temperature on the growth and development of the early-weaned piglet 2. Energy metabolism. Anim. Sci..

[16] Baxter E.M., Jarvis S., Sherwood L., Farish M., Roehe R., Lawrence A.B., Edwards S.A. (2011). Genetic and environmental effects on piglet survival and maternal behaviour of the farrowing sow. Appl. Anim. Behav. Sci..

[17] Titterington R.W., Fraser D. (1975). The lying behaviour of sows and piglets during early lactation in relation to the position of the creep heater. Appl. Anim. Ethol..

[18] Zhou H., Xin H., Bundy D.S. (1996). Dynamic thermoregulatory behaviors of neonatal piglets exposed to heat lamps. ASABE Pap..

[19] Hrupka B.J., Leibbrandt V.D., Crenshaw T.D., Benevenga N.J. (1998). The effect of farrowing crate heat lamp location on sow and pig patterns of lying and pig survival. J. Anim. Sci..

[20] Xin H., Zhou H., Bundy D.S. (1997). Comparison of energy use and piglet performance between conventional and energy-efficient heat lamps. Appl. Eng. Agric..

[21] Zhang J., Liu H., Zheng P. (2021). Design and experiment of intelligent control system for local temperature of suckling piglets. J. Domest. Anim. Ecol..

[22] Zheng P., Zhang J., Liu H., Bao J., Xie Q., Teng X. (2021). A wireless intelligent thermal control and management system for piglet in large-scale pig farms. Inf. Process. Agric..

[23] Yu M., Wu P., Han D. (2012). Design and experimental research on auto-control system of temperature in piglets house based on PLC. J. Agric. Mech. Res..

[24] de Souza Granja Barros J., Rossi L.A., Sartor K. (2016). PID temperature controller in pig nursery: Improvements in performance, thermal comfort, and electricity use. Int. J. Biometeorol..

[25] Zhou X.R., Jiang S., Yang F.Y., Xiao R., Liu Z.H. (2019). Application effect of a weaned piglet incubator designed using far infrared carbon fiber heating plate. Anim. Husb. Vet. Med..

[26] Pig Improvement Company (2016). Gilt and Sow Management Guidelines.

[27] (2008). Environmental Parameters and Environmental Management for Intensive Pig Farms.

[28] Gong J., He Z., Lei Y., Yang L., Zeng K., Tao X., Wan Z. (2015). Study on the Spatiotemporal Distribution Patterns of Environmental Parameters in Traditional Farrowing Houses. Swine Prod..

[29] Xie Q.J., Zheng P., Bao J., Su Z.B. (2020). Thermal environment prediction and validation based on deep learning algorithm in closed pig house. Trans. Chin. Soc. Agric. Mach..

[30] Yang Y., Jian G., Li R., Wang Y.Q., Pang C., Zhao S.G., Zhang J., Li X.S. (2021). Study on the Spatial Requirements of Heating Areas for Piglets in Large-Scale Pig Farms. Anim. Sci. Abroad (Pigs Poult.).

[31] Ziron M., Hoy S. (2003). Effect of a warm and flexible piglet nest heating system—The warm water bed—On piglet behaviour, live weight management and skin lesions. Appl. Anim. Behav. Sci..

[32] Hill W.G. (2014). Applications of population genetics to animal breeding, from Wright, Fisher and Lush to genomic prediction. Genetics.

